# Therapeutic response and long-term outcome of differentiated thyroid cancer with pulmonary metastases treated by radioiodine therapy

**DOI:** 10.18632/oncotarget.21570

**Published:** 2017-10-06

**Authors:** Jing Yang, Meng Liang, Yingying Jia, Li Wang, Lin Lin, Jianhua Geng, Shengzu Chen, Ye-Xiong Li, Rong Zheng

**Affiliations:** ^1^ Department of Nuclear Medicine, National Cancer Center/Cancer Hospital, Chinese Academy of Medical Sciences and Peking Union Medical College, Beijing 100021, China; ^2^ Department of Radiation Oncology, National Cancer Center/Cancer Hospital, Chinese Academy of Medical Sciences and Peking Union Medical College, Beijing 100021, China

**Keywords:** thyroid cancer, pulmonary metastases, radioiodine therapy

## Abstract

**Objective:**

To explore the therapeutic response (TR) and long-term outcomes of iodine-131 (I-131) treatment for patients with differentiated thyroid cancer and pulmonary metastases (DTC+PM), as well as the association between the assessment of TR and long-term outcomes.

**Methods:**

This retrospective study comprised 151 DTC+PM patients. TR was evaluated by changes in serum levels of thyroglobulin, anatomic imaging and iodine uptake in pulmonary nodules; logistic regression was applied to identify predictors. Overall survival (OS) was calculated using the Kaplan–Meier method and predictive factors of outcome by multivariate analyses.

**Results:**

After I-131 treatment, 17 patients achieved a complete response, 71 a partial response, and 63 no response. Age, pulmonary nodule size, iodine-concentration within PM, extra-PM, frequency and cumulative dose of I-131 treatment were significant for TR. OS was 72.2% at 5, 55.2% at 10 and 51.3% at 15 years. After adjustment for other factors, age, pulmonary nodule size, extra-PM, frequency and cumulative dose of I-131 treatment were significant. A significant difference of survival rate in patients with different TR group was observed.

**Conclusions:**

There was a supportive response and prognosis for I-131 treatment upon DTC+PM patients. Older patients and those with non-I-131-avid PM were more likely to have no response to I-131 treatment, and greater benefits could be achieved by patients who complete treatment. Long-term outcome was better in patients with age <45 years, pulmonary nodule size <2 cm, without extra-PM, and the frequency of iodine treatment ≥5 times. The predictive power of the TR on long-term prognosis was favorable.

## INTRODUCTION

Differentiated thyroid cancer (DTC) is one of the most curable endocrine cancers and is usually associated with an excellent prognosis [1]. Distant metastases, which occur in 2.2–12.4% of patients with known DTC, are the most common cause of death in these patients [2–8]. The lungs are the most common sites of distant metastases in DTC, and patients with pulmonary metastases (PM), in general, have a poor prognosis. About 40% of DTC patients with PM die within 10 years [9]. Treatment options for DTC are surgery, radioiodine therapy (RAIT) and suppression of thyroid hormones, and long-term monitoring after treatment is essential [10]. Management for DTC patients with PM is based on thyroidectomy, followed by radioiodine therapy and subsequent hormone-replacement therapy [10]. RAIT has been a standard adjuvant treatment after total or near-total thyroidectomy [11, 12].

Several studies have reported that iodine-131 (I-131)-avid PM are curable and are associated with an excellent prognosis. In contrast, non-I-131-avid PM have a relatively poor prognosis and are associated with high thyroglobulin (Tg) levels [9, 13]. However, few studies have clarified the factors influencing the response of I-131 treatment and the prognostic factors influencing the long-term survival for DTC patients with PM (especially for patients with I-131-avid PM)[14, 15].

The present study was designed to select DTC patients with PM for RAIT in China. Data from a clinical database on long-term follow-up for DTC patients with PM were analyzed retrospectively. We aimed to: (i) assess the therapeutic response of RAIT on DTC patients with PM; (ii) evaluate the overall survival (OS) and related variables; (iii) explore the association between the therapeutic response and long-term prognosis, and then assess if the criteria used for the assessment of therapeutic response were reliable.

## RESULTS

### Characteristics of patients with pulmonary metastases

The clinical characteristics of the 151 DTC patients with PM were summarized in Table 1. Among the 151 patients, 86 were women and 65 were men (male:female ratio = 1:1.32) and 73.5% had PTC. PM alone were involved in 124 patients, whereas 27 patients also had metastases to other organs. All 151 patients underwent thyroidectomy: 82 patients received unilateral total and contralateral subtotal thyroidectomy first and then underwent a second procedure to remove remaining thyroid tissue; total thyroidectomy was done as the primary procedure in 69 cases. I-131-avid PM were observed in 122 patients and non-I-131-avid PM were found in 29 patients. Of the 151 patients diagnosed with PM, 77 patients had a cumulative dose 19.6 GBq (530 mCi) mCi whereas the others had doses <19.6 GBq (530 mCi).

**Table 1:** Clinical characteristics of patients with differentiated thyroid cancer and pulmonary metastases and the response to radioiodine therapy

Factor	No. of patientsNo. (%)	CR	PR	NR	P
Sex					0.634
Male	65 (43.0)	7	28	30	
Female	86 (57.0)	10	43	33	
Age at PM diagnosis (years)					<0.001
<45	66 (43.7)	13	43	10	
≥45	85 (56.3)	4	28	53	
Pathology					0.727
PTC	111 (73.5)	14	52	45	
FTC	40 (26.5)	3	19	18	
Capsule invasion					0.016
Yes	66 (43.7)	5	27	34	
No	9 (6.0)	1	8	0	
Unknown	76 (50.3)	11	36	29	
Pulmonary nodule size (cm)					0.055
<1	67 (44.4)	12	35	20	
≥1	46 (30.4)	3	19	24	
Unknown	38 (25.2)	2	17	19	
PM type					0.002
Diffuse	131 (86.7)	10	67	54	
Focal	14 (9.3)	6	3	5	
Unknown	6 (4.0)	1	1	4	
Tg level					0.010
High (>10 mg/L)	112 (74.2)	8	59	45	
Normal (≦10 mg/L)	39 (25.8)	9	12	18	
Iodine concentration within PM					<0.001
Yes	122 (80.8)	17	67	38	
No	29(19.2)	0	4	25	
Extra-PM					0.015
Yes	27(17.9)	0	10	17	
No	124 (82.1)	17	61	46	
Frequency of I-131 treatment					0.053
<5	96 (63.6)	10	39	47	
≥5	55 (36.4)	7	32	16	
Cumulative dose (mCi)					0.010
<530	74 (49.0)	7	27	40	
≥530	77 (51.0)	10	44	23	
Radiotherapy or chemotherapy					0.105
Yes	30 (19.9)	4	9	17	
No	121 (80.1)	13	62	46	

CR: complete response; PR: partial response; NR: no response; PTC: papillary thyroid carcinoma; FTC: follicular thyroid carcinoma; Tg: thyroglobulin; PM: pulmonary metastases.

### Therapeutic response and influencing factors

The therapeutic response of 151 DTC patients with PM to RAIT was shown in Table 1. Of the 151 patients, 17 achieved a CR (11.3%), 71 PR (47.0%) and 63 NR (41.7%). Age, capsule invasion, metastases type, Tg level, iodine concentration within PM, extra-PM, and the cumulative dose of 131-I treatment in the three therapeutic-response groups were significantly different (P < 0.001, 0.016, 0.002, 0.010, < 0.001, 0.015, and 0.010, respectively).

Univariate logistic regression analyses of the factors influencing the therapeutic response were shown in Table 2. Differences in age, size of pulmonary nodules, iodine concentration within PM, extra-PM, frequency and the cumulative dose of I-131 treatment were significant (P < 0.001, 0.032, < 0.001, 0.016, 0.018, and 0.003, respectively). Age <45 years and I-131-avid PM had the most favorable effect on therapeutic response, and the relative ratios were 9.28 (95% confidence interval (CI): 4.15–20.71) and 13.82 (95% CI: 4.50–42.47), respectively.

**Table 2:** Univariate analyses of the therapeutic response and influencing factors in 151 patients with differentiated thyroid cancer and pulmonary metastases

Factor	No. of patients	OR	NR	P	Relative ratio (95% CI)
Age at PM diagnosis (years)				<0.001	9.28 (4.15–20.71)
≥45	85	32	53		
<45	66	56	10		
Pulmonary nodule size (cm)				0.032	
Unknown	38	19	19	0.042	2.35 (1.03–5.35)
≥1	46	22	24	0.018	2.56 (1.18–5.59)
<1	67	47	20		
Iodine concentration within PM				<0.001	13.82 (4.50–42.47)
No	29	4	25		
Yes	122	84	38		
Extra-PM				0.016	2.88 (1.22–6.83)
Yes	27	10	17		
No	124	78	46		
Frequency of I-131 treatment				0.018	2.34 (1.15–4.74)
<5	96	49	47		
≥5	55	39	16		
Cumulative dose (mCi)				0.003	2.76 (1.42–5.39)
<530	74	34	40		
≥530	77	54	23		

OR: overall response; NR: no response; PM: pulmonary metastases.

### Survival and prognostic survival factors

**Figure 1:**
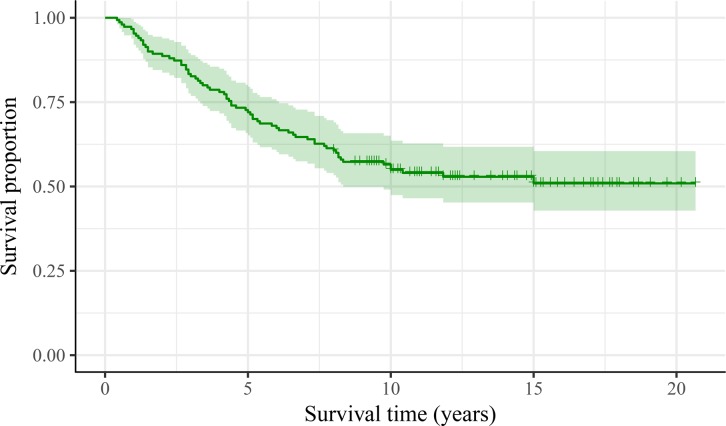
Survival curves form the diagnoses of pulmonary metastases for 151 patients with differentiated thyroid cancer and pulmonary metastases

OS from the diagnoses of PM were shown in Figure 1. Prognostic survival factors were listed in Table 3. Median survival of the 151 patients was 9.6 years, and OS was 72.2% at 5 years, 55.2% at 10 years and 51.3% at 15 years, respectively. OS at 10 years was 90.9% in patients aged <45 years and 18.8% in patients aged ≥45. For patients with PM only, survival at 10 years was 65.1%, which was significantly higher than patients with extra-PM (9.1%). OS at 10 years was 78.8% in patients with micro-nodular metastases (<1 cm) and 27.7% in those with macro-nodular metastases (≥1 cm).

**Table 3:** Univariate and multivariate survival analyses for 151 patients with differentiated thyroid cancer and pulmonary metastases

Factor	No. of patients	5-year OS (%)	10-year OS (%)	15-year OS (%)	Univariate P	Multivariate P	Hazard ratio (95% CI)
Age at PM diagnosis					<0.001	<0.001	8.55 (3.53–20.73)
≥45	85	31.6	18.8	18.8			
<45	66	92.4	90.9	90.9			
Pathology					<0.001		
PTC	111	79.3	64.4	58.8			
FTC	40	52.5	29.8	29.8			
Capsule invasion					0.007		
Unknown	76	73.7	61.8	60.1			
No	9	88.9	88.9	88.9			
Yes	66	66.7	46.4	34.5			
Pulmonary nodule size (cm)					<0.001	0.018	
Unknown	38	65.8	47.1	43.4		0.045	2.00 (1.02–3.95)
≥1	46	56.5	27.7	24.6		0.005	2.58 (1.34–4.98)
<1	67	86.6	78.8	73.9			
Iodine concentration in PM					0.007		
Yes	122	74.6	60.3	55.9			
No	29	62.1	33.2	33.2			
Extra-PM					<0.001	0.007	2.11 (1.23–3.63)
Yes	27	40.7	9.3	0			
No	124	79.0	65.1	65.1			
Frequency of I-131 treatment					<0.001	0.002	2.55 (1.40–4.63)
<5	96	61.5	44.4	42.6			
≥5	55	90.9	74.1	67.2			
Cumulative dose (mCi)					0.003		
<530	74	60.8	44.2	41.3			
≥530	77	83.1	65.8	60.9			
Therapeutic response					<0.001		
CR	17	100.0	100.0	100.0			
PR	71	83.1	74.4	72.4			
NR	63	52.4	21.2	0.0			

OS: overall survival; CR: complete response; PR: partial response; NR: no response; PTC: papillary thyroid carcinoma; FTC: follicular thyroid carcinoma; PM: pulmonary metastases.

**Figure 2:**
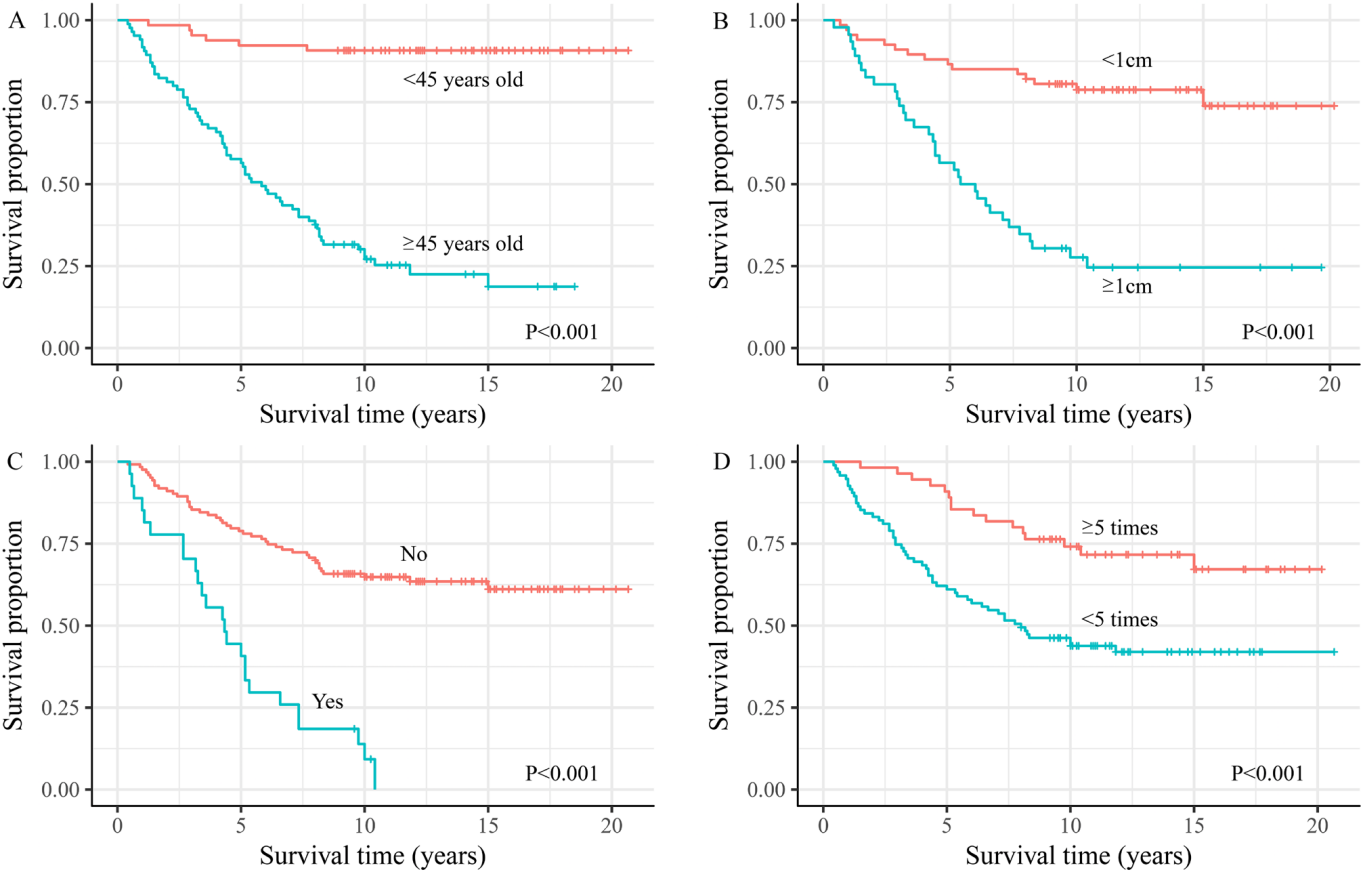
Overall survival time from diagnosis of pulmonary metastases for the differentiated thyroid cancer with pulmonary metastasis patients by **(A)** age at diagnosis of metastasis, **(B)** pulmonary nodules size, **(C)** extra pulmonary metastasis, and **(D)** frequency of I-131 treatment.

For all 151 patients, univariate analyses showed that remarkably poor survival outcomes were associated with patients: aged ≥45 years; with FTC; with macro-nodular metastases; with non-I-131-avid PM; with capsule invasion; with extra-PM; who had undergone I-131 treatment frequency fewer than five times or who had a cumulative dose <530 mCi. After adjustment for other significant factors, Cox's regression analyses showed that age, size of pulmonary nodules, combination with extra-PM, frequency of I-131 treatment remained significant for survival (P < 0.001, 0.018, 0.007 and 0.002, respectively) (Figure 2).

### Patients with I-131 avid pulmonary metastases and their predictive factors

I-131 non-avid PM was the most predictable variable for a poor outcome. Hence, we extracted all 122 patients with I-131-avid PM and a subset analysis of therapeutic response as well as survival outcome was conducted to determine which factors contributed to the outcome of I-131 treatment for DTC patients with I-131-avid PM.

For all 122 patients with I-131-avid PM, the associations between therapeutic response and age and extra-PM remained significant (P < 0.001 and 0.007, respectively) whereas the other factors were no longer significant. As expected, the estimate of Cox's regression model showed a similar pattern for all 151 DTC patients, and the hazard ratios were slightly different from the predicted age (8.55 to 10.46), size of pulmonary nodules (2.58 to 3.14), extra-PM (2.11 to 2.17) and frequency of I-131 treatment (2.55 to 3.12) (Table 3 and Table 4).

**Table 4:** Univariate and multivariate survival analyses for 122 differentiated thyroid cancer patients with I-131-avid pulmonary metastases

Factors	No. of patients	5-year OS (%)	10-year OS (%)	15-year OS (%)	Univariate P	Multivariate P	Hazard ratio (95% CI)
Age at PM diagnosis (years)					<0.001	<0.001	10.46 (3.94–27.74)
≥45	60	55.00	27.5	18.7			
<45	62	93.50	91.9	91.9			
Pathology					<0.001		
PTC	87	82.20	70.9	69.1			
FTC	35	53.10	30.9	17.7			
Capsule invasion					0.006		
Unknown	76	76.6	67.2	65.3			
No	9	100.0	100.0	100.0			
Yes	66	68.6	45.5	36.5			
Pulmonary nodule size					<0.001	0.016	
Unknown (cm)	32	65.60	50.0	45.8		0.087	1.95 (0.91–4.21)
≥1	32	56.30	33.8	29.5		0.004	3.14 (1.44–6.88)
<1	58	89.70	80.7	75.7			
Extra-PM					<0.001	0.015	2.17 (1.16–4.04)
Yes	21	38.10	9.5	0			
No	101	82.20	71.2	67.2			
Frequency of I-131 treatment					0.002	0.001	3.12 (1.58–6.18)
<5	69	60.9	49.0	46.9			
≥5	53	92.5	75.1	68.0			
Cumulative dose (mCi)					0.007		
<530	50	60.00	47.5	43.9			
≥530	72	84.70	69.2	64.1			
Therapeutic response					<0.001		
CR	17	100.0	100.0	100.0			
PR	67	82.1	74.3	72.3			
NR	38	50.0	17.5	0.0			

OS: overall survival; CR: complete response; PR: partial response; NR: no response; PTC: papillary thyroid carcinoma; FTC: follicular thyroid carcinoma; PM: pulmonary metastases.

### Association of the therapeutic response and long-term prognosis

**Figure 3:**
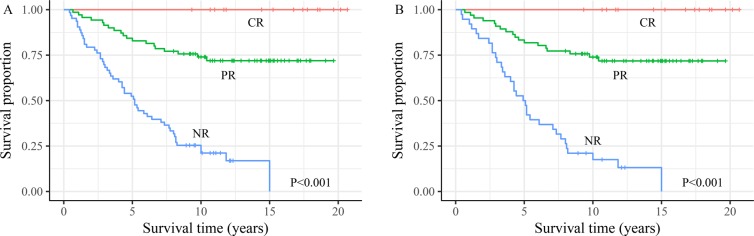
The survival analysis of differentiated thyroid cancer with pulmonary metastasis patients for three initial therapy risk response groups involved in **(A)** all 151 differentiated thyroid cancer with pulmonary metastases patients and **(B)** 122 I-131-avid patients.

We wished to: (i) evaluate the criteria for assessment of the therapeutic response for DTC patients with PM; (ii) discover if an assessment of therapeutic response during I-131 treatment could modify these initial risk estimates. Hence, two targeted survival analyses were carried out (Figure 3).

For all 151 DTC patients with PM, OS at 15 years for the 17 patients with a CR was 100%. In contrast, OS was 72.4% for the 71 patients with a PR and 0% for the 63 patients with NR. A similar pattern was observed for the 122 patients with I-131-avid PM, patients with a CR had a better prognosis than those with a PR, whereas the worst prognosis was observed in the 38 patients with NR. All differences among the three groups were significant (P < 0.001).

## DISCUSSION

In this retrospective study of 151 DTC patients with PM, 11.3% and 47.0% patients achieved a CR and PR, respectively. Results suggested that older and patients with non-I-131-avid PM were more likely to show no response to initial I-131 treatment, and that greater benefits could be achieved by patients with I-131-avid PM who adhered to completion of I-131 treatment. 70 patients died during the follow-up period, and 81 patients survived or were lost to follow-up, resulting in OS at 5, 10 and 15 years of 72.2%, 55.2% and 51.3% respectively. Multivariate analyses showed that better long-term outcome was related to: (i) clinical factors (age at the diagnosis of PM <45 years, size of PM <2 cm, without extra-PM); (ii) treatment factors (frequency or the cumulative dose of I-131 treatment ≥5 times). In addition, there were significant differences (P < 0.001) in long-term OS among patients with different therapeutic responses.

Only 58.3% patients achieved a CR or PR, which was lower than that reported in other studies using identical criteria in China [9, 14, 16–18]. The reason underlying this low prevalence for a CR may have been: (i) more than half of patients were aged ≥45 years; (ii) 92.7% of patients were diagnosed with PM by anatomic imaging; (iii) most patients (85/151) were diagnosed as having advanced DTC according to the NCCN (National Comprehensive Cancer Network) guidelines.

OS was consistent with that in a study in Shanghai (China)[9], higher than that in studies undertaken in western countries [19, 20], and lower than that in other Asian studies [3, 21]. Early diagnosis of thyroid cancer in Asia (especially in South Korea and China) in recent years could explain the favorable long-term prognosis in Asian countries [22, 23].

One of the main aims of this study was to evaluate the criteria for assessment of the therapeutic response for DTC patients with PM, as excepted, a supportive result was observed. Survival analyses showed significant differences (P < 0.001) in long-term survival among patients with different therapeutic responses. Patients who had a CR had better prognosis than those who had a PR, whereas the worst prognosis was observed for those with a NR. In our study, the change in serum levels of Tg and anatomic imaging were considered to be cooperative prognostic indicators for assessment of the response to I-131 treatment for PM. Numerous studies have been done on the prognostic value for disease-free remission and death of DTC patients [24–26]. It is thought that the serum level of Tg under TSH increase (>30 uIU/ml) is the most reliable indicator for persistent/recurrent disease and remission, and smaller nodules in anatomic imaging is related to a favorable response to I-131 treatment [9, 14, 16–18]. Given those facts, the criteria for assessment of the therapeutic response for DTC patients with PM was acceptable and the predictive power of the TR on long-term prognosis was favorable.

Age has been demonstrated to predict the response and mortality risk of I-131 treatment in DTC patients with PM [9, 13, 17]. We also found age to be a significant predictor, with a risk of NR increasing 6.0% per year. After adjustment for pulmonary nodule size, extra-PM, and the frequency of I-131 treatment, the mortality risk in DTC patients with PM increased by 6.3% per year. This result was consistent with those of Huang *et al.*,[3] Schlumberger *et al.*, [27] and Ruegemer *et al.*,[28] with mortality risk increasing by 5.6%, 4% and 7% per year, respectively.

The size of pulmonary lesions (macro- or micro-nodules) was an important prognostic factor. Favorable long-term outcomes were observed for patients with smaller metastases. Macro-nodular metastases showed poor iodine concentration within PM (14/46, 30.4%) and poor OS. Song *et al.*[9] showed that micro-nodular metastases were susceptible to I-131 treatment and had a good prognosis, whereas macro-nodular metastases of non-I-131-avid nodules had a poor prognosis. The prevalence of a CR for DTC patients with PM undetected by CT of the chest was 23.8% and the OR was 95.2%[8], which were higher than those of studies involving CT-based diagnoses of patients [9, 14, 16–18]. This result may have been because patients with larger nodules are more likely to have a later stage of DTC, a poorer concentration of iodine within metastases, and only a small number of patients are cured [8, 9].

PTC was more common than FTC in patients with PM. However, FTC was shown to have a negative impact on long-term survival for DTC patients with PM, as demonstrated by other scholars [17, 29]. FTC is an aggressive type of malignancy and several extra-pulmonary organs or sites can be invaded by hematogenous metastases. Extensive researches suggested that FTC is prone to distant metastases compared with PTC [8, 17].

The difference in long-term outcome between patients with pulmonary-only metastases and those with extra-PM was significant (P < 0.001). Studies have shown that bone metastases have a worse curative effect than that of PM with regard to I-131 treatment in DTC patients with PM [15, 20]. This difference may be because I-131 treatment results in a better prognosis for DTC patients with PM than those for patients with bone metastases or other distant metastases [15, 29], and multiple metastases often demonstrate advanced thyroid cancer.

The long-term prognosis of patients with iodine concentration within PM was better than those without iodine concentration within PM. Non-I-131-avid metastases often demonstrate dedifferentiated thyroid cancer cells, which is accompanied by reduced expression of the sodium iodide symporter, the TSH receptor, and thyroid peroxidase [30–32]. Several studies have shown very limited benefit by RAIT in patients with non-I-131-avid pulmonary disease [20, 21, 33].

For all 151 patients, having I-131 treatment ≥5 occasions or a cumulative dose ≥530 mCi elicited a better therapeutic response and long-term outcome. Our result might have been because patients with non-I-131-avid PM usually resorted to other treatment options. Only 6.9% of patients had more than five courses of I-131 treatment, which resulted in poor long-term outcomes in those patients. We further analyzed the survival prognostic factors of 122 patients with I-131-avid metastases separately; univariate and multivariate analyses were consistent with the results of the analyses of all 151 patients. This observation suggested that I-131 treatment was beneficial for the long-term outcome of DTC patients with PM, and that patients with I-131-avid metastases should complete all courses of treatment.

Our study had several limitations. First, because it was retrospective and included only those DTC patients treated by RAIT in a single center in China, the results might be derived from a biased patient group. Second, during the long study period, small portion of clinical data were missing, which might have caused bias in data selection. Third, the pathologic diagnosis of PM was not always available, so it was diagnosed based on imaging and increased serum levels of Tg. However, the detection of thyroglobulin antibody (TgAb) was not always available in study period in our hospital, this may potentially affect the accurate evaluation of therapeutic response. In addition, we could not completely rule out the possibility of other conditions, such as incidental primary lung cancers or benign lung disorders such as inflammation.

Despite these limitations, the results of this study may have important clinical implications. These data suggested that older patients and those with non-I-131-avid PM were more likely to have NR to initial I-131 treatment. Also, greater benefits could be achieved upon completion of I-131 treatment for DTC patients with I-131-avid PM. Long-term outcome was related to age, the size of PM, whether PM were accompanied by other distant metastases, and the frequency of RAIT. Moreover, by accessing the therapeutic response to I-131 therapy, clinicians can have an insight into the long-term prognosis for DTC patients with PM. All of these may be helpful to guide clinical treatment for DTC with PM patients.

## MATERIALS AND METHODS

### Patient eligibility

This retrospective cohort yielded 151 DTC with PM patients at the department of nuclear medicine between February 1991 and July 2007. The median ages at the diagnosis of DTC and PM were 42 (range, 6–82) and 49 (range, 11–86) years, respectively. One-hundred and eleven patients were diagnosed as having papillary thyroid carcinoma (PTC) and 40 patients as having follicular thyroid carcinoma (FTC). PM only were involved in 124 patients, whereas 27 patients also had distant metastases to other organs.

### Diagnostic of DTC pulmonary metastases

A patient satisfying the following criteria was considered to have PM: (i) pathology results confirming PM; (ii) I-131 whole-body scan (I-131-WBS) demonstrating iodine concentration within unilateral lung or bilateral lungs; (iii) positive anatomic imaging (radiography, computed tomography (CT) of the chest).

### I-131 Treatment protocols and evaluation of therapeutic response

All 151 patients underwent thyroidectomy. Neck dissection was undertaken if any lymph node was enlarged and malignancy was suspected according to preoperative imaging or physical examination. All patients were instructed to follow a low-iodine diet for ≥2 weeks. During the 3–4 weeks before the start of I-131 treatment, all patients were forbidden to take any iodine or iodine substance, and withdrawal of thyroid hormones had achieved a sufficient hypothyroid state (serum level of thyroid-stimulating hormone (TSH) >30 uIU/ml. Conventional measurements, including those for free tri-iodothyronine, free thyroxine, TSH, Tg, neck ultrasonography and CT of the chest, were done before I-131 administration. All 151 patients were treated with thyroid hormone therapy from the day after I-131 treatment. I-131-WBS was carried out 7 days after oral administration of I-131 to observe I-131 uptake by PM. After surgery, all patients received one dose of 2.96–5.55 GBq (80–150 mCi) RAIT (median dose was 3.70 GBq, dose was halved for children) to remove residual thyroid tissue. The therapeutic dose was based on a fixed dose method combined with maximum dose experience, a single dose of RAIT for PM was 2.96–7.4 GBq (80–200 mCi) with the median dose of 5.55 GBq (150 mCi), and the median cumulative RAIT dose was 19.6 GBq (530 mCi). The indication of stopping treatment in non-I-131-avid PM was as follow: after the low iodine diet and elevating thyroid stimulating hormone TSH, the WBS imaging after RAI treatment showed that the lung metastasis did not take iodine, and the lung metastasis in CT imaging increased or enlarged.

Based on imaging (including WBS) and serum levels of Tg [8], therapeutic response was classified as: (i) complete response (CR)—no symptoms of PM, no abnormal concentration of iodine within PM upon I-131-WBS or other imaging examinations, and Tg negativity (serum Tg <1 ng/mL with TSH stimulation); (ii) partial response (PR)—concentration of iodine within PM reduced in I-131-WBS and metastatic pulmonary nodules were smaller or less in other imaging examinations, with reduced Tg levels (compared with inhibition or stimulation of TSH). (iii) no response (NR)—no improvement or increased concentration of iodine within PM and no change or enlargement or more of metastatic pulmonary nodules in other imaging examinations, with increased Tg levels. The overall response (OR) was CR plus PR.

### Follow-up and statistical analyses

All patients were observed at follow-up even if they were admitted to other departments or hospitals. Patients who completed the entire treatment in our hospital were followed up at outpatient clinics annually for first 5 years and then every 2 or 3 years. Patients transferred to other hospitals were followed up by telephone. This was a study looking at long-term outcome, so all patients had been followed up ≥5 years after the diagnosis of metastases. Median follow-up was 11.2 (range, 0.4–20.5) years. Statistical analyses were undertaken on follow-up data collected up to September 2015 or at the time of death resulting from any cause. OS was calculated from the initial diagnosis of PM until the time of death resulting from any cause or until the final follow-up assessment.

The chi-square or Fisher's exact test was used to estimate the differences between groups. A binary logistic regression model was fitted to assess which factors contributed to the outcome of response to I-131 treatment. Kaplan–Meier survival analyses were applied to evaluate the effect of each potential prognostic variable on survival, and the differences between groups were compared using the log-rank test. Multivariate analyses were done with a Cox proportional hazards model to assess the relationship between survival time and several variables simultaneously. The patients were divided into three groups according to their therapeutic responses, after long-term follow-up, the differences of survival curves in 3 groups were tested to access whether the evaluation of the therapeutic response was reliable. P<0.05 was considered significant. Data were analyzed using R v3.2.5 (R Foundation for Statistical Computing).

## Abbreviations

TR: therapeutic response
PM: pulmonary metastases
OS: overall survival
DTC: differentiated thyroid cancer
RAIT: radioiodine therapy
Tg: thyroglobulin
PTC: papillary thyroid carcinoma
FTC: follicular thyroid carcinoma
CT: computed tomography
WBS: whole-body scan
TSH: thyroid-stimulating hormone
CR: complete response
PR: partial response
NR: no response
OR: overall response
CI: confidence interval
TgAb: thyroglobulin antibody.
